# Genome Analysis of a Novel *Bradyrhizobium* sp. DOA9 Carrying a Symbiotic Plasmid

**DOI:** 10.1371/journal.pone.0117392

**Published:** 2015-02-24

**Authors:** Shin Okazaki, Rujirek Noisangiam, Takashi Okubo, Takakazu Kaneko, Kenshiro Oshima, Masahira Hattori, Kamonluck Teamtisong, Pongpan Songwattana, Panlada Tittabutr, Nantakorn Boonkerd, Kazuhiko Saeki, Shusei Sato, Toshiki Uchiumi, Kiwamu Minamisawa, Neung Teaumroong

**Affiliations:** 1 Graduate School of Agriculture, Tokyo University of Agriculture and Technology, Tokyo, Japan; 2 School of Biotechnology, Institute of Agricultural Technology, Suranaree University of Technology, Nakhon Ratchasima, Thailand; 3 Graduate School of Life Science, Tohoku University, Sendai, Japan; 4 Faculty of Life Sciences, Kyoto Sangyo University, Motoyama, Kamigamo, Kita-Ku, Kyoto 603-8555, Japan; 5 Center of Omics and Bioinformatics, Graduate School of Frontier Sciences, University of Tokyo, Tokyo, Japan; 6 Department of Biological Sciences, Faculty of Science, Kyousei Science Center for Life and Nature, Nara Women’s University, Kitauoya Nishimachi, Nara 630-8506, Japan; 7 Graduate School of Science and Engineering, Kagoshima University, Kagoshima, Japan; Instituto Butantan, BRAZIL

## Abstract

*Bradyrhizobium* sp. DOA9 isolated from the legume *Aeschynomene americana* exhibited a broad host range and divergent nodulation (*nod*) genes compared with other members of the *Bradyrhizobiaceae*. Genome analysis of DOA9 revealed that its genome comprised a single chromosome of 7.1 Mbp and a plasmid of 0.7 Mbp. The chromosome showed highest similarity with that of the *nod* gene-harboring soybean symbiont *B. japonicum* USDA110, whereas the plasmid showed highest similarity with pBBta01 of the *nod* gene-lacking photosynthetic strain BTAi1, which nodulates *Aeschynomene* species. Unlike in other bradyrhizobia, the plasmid of DOA9 encodes genes related to symbiotic functions including nodulation, nitrogen fixation, and type III/IV protein secretion systems. The plasmid has also a lower GC content (60.1%) than the chromosome (64.4%). These features suggest that the plasmid could be the origin of the symbiosis island that is found in the genome of other bradyrhizobia. The *nod* genes of DOA9 exhibited low similarity with those of other strains. The *nif* gene cluster of DOA9 showed greatest similarity to those of photosynthetic bradyrhizobia. The type III/IV protein secretion systems of DOA9 are similar to those of *nod* gene-harboring *B. elkanii* and photosynthetic BTAi1. The DOA9 genome exhibited intermediate characteristics between *nod* gene-harboring bradyrhizobia and *nod* gene-lacking photosynthetic bradyrhizobia, thus providing the evidence for the evolution of the *Bradyrhizobiaceae* during ecological adaptation. *Bradyrhizobium* sp. DOA9 isolated from the legume *Aeschynomene americana* exhibited a broad host range and divergent nodulation (*nod*) genes compared with other members of the *Bradyrhizobiaceae*. Genome analysis of DOA9 revealed that its genome comprised a single chromosome of 7.1 Mbp and a plasmid of 0.7 Mbp. The chromosome showed highest similarity with that of the *nod* gene-harboring soybean symbiont *B. japonicum* USDA110, whereas the plasmid showed highest similarity with pBBta01 of the *nod* gene-lacking photosynthetic strain BTAi1, which nodulates *Aeschynomene* species. Unlike in other bradyrhizobia, the plasmid of DOA9 encodes genes related to symbiotic functions including nodulation, nitrogen fixation, and type III/IV protein secretion systems. The plasmid has also a lower GC content (60.1%) than the chromosome (64.4%). These features suggest that the plasmid could be the origin of the symbiosis island that is found in the genome of other bradyrhizobia. The *nod* genes of DOA9 exhibited low similarity with those of other strains. The *nif* gene cluster of DOA9 showed greatest similarity to those of photosynthetic bradyrhizobia. The type III/IV protein secretion systems of DOA9 are similar to those of *nod* gene-harboring *B. elkanii* and photosynthetic BTAi1. The DOA9 genome exhibited intermediate characteristics between *nod* gene-harboring bradyrhizobia and *nod* gene-lacking photosynthetic bradyrhizobia, thus providing the evidence for the evolution of the *Bradyrhizobiaceae* during ecological adaptation.

## Introduction

The interaction between leguminous plants and rhizobia results in the formation of root nodules. Within legume nodules rhizobia fix atmospheric nitrogen to ammonia, which can support the growth of host plants. Rhizobia possess a set of modulation (*nod*) genes that control the synthesis of lipochitooligosaccharides called nod factors (NFs) [1], which induce signal transduction cascades in host plants, ultimately leading to nodule formation [1]. The rhizobial *nod* genes were previously thought to be essential for nodule formation; however, the recent discovery of *Bradyrhizobium* spp. ORS278 and BTAi1, which lack *nod* genes, but are able to nodulate some species of the semi-aquatic legume genus *Aeschynomene*, contradicts this long-held dogma [2].

*Aeschynomene* species can be divided into two groups according to the mechanism of nodule initiation. One group, including *A*. *afraspera* and *A*. *americana*, initiates nodulation through NFs, whereas the other group, including *A*. *indica* and *A*. *sensitiva*, initiate**s** nodulation without NFs. Although the genome of some *Aeschynomene* symbionts including ORS278 and BTAi1 were sequenced [2], the genetic basis for NF-independent nodulation and the evolutionary relationship between NF-independent and NF-dependent nodulation remains mostly unclear [2,3].

A recent interest in the origin and evolution of *Bradyrhizobiaceae* rhizobia has developed because the genomes of several strains have been sequenced. The genome analyses of *B*. *japonicum* USDA110 [4] and *B*. *japonicum* USDA6 ^T^ [5] revealed that genes for nodule symbiosis, such as *nod*, for nitrogen fixation, and for secretion systems are located on integrated genomic islands (symbiosis islands), suggesting that the symbiotic genes were acquired by lateral gene transfer from an unknown donor. This evolution scenario is supported by the detailed genome comparison between symbiotic (*B*. *japonicum* USDA110) and nonsymbiotic (*Bradyrhizobium* sp. S23321) bradyrhizobia [6]. However, the origin and transmission mechanism of symbiotic genes remain**s** poorly understood.

We recently isolated several strains of bradyrhizobia from the root nodules of *A*. *americana* [7]. One isolate, *Bradyrhizobium* sp. DOA9, was found to nodulate a broad range of leguminous hosts, including dalbergioid, millettioid, and robinoid trives and was also found to be an endophyte of rice, although DOA9 did not form nodules on NF-independent group**s** of *Aeschynomene* [8]. DOA9 possesses highly divergent *nod* genes that could not be amplified by general primer sets designed on other bradyrhizobia [7]. Furthermore, Southern blot hybridization suggested that some of the *nod* genes and nitrogen fixation (*nif*) genes of DOA9 are localized on the plasmid unlike in other bradyrhizobia [8]. These results suggested that DOA9 possesses a novel type of genome with *nod* genes divergent from previously reported bradyrhizobia.

Here we analyzed the genome of DOA9 and compared it with the genomes of other bradyrhizobia. Several symbiosis-related genes, including those involved in nodulation, nitrogen fixation, and secretion systems, were investigated in order to highlight the genetic basis of the broad host range of DOA9 and the evolutionary relationships between *nod* gene-harboring soybean bradyrhizobia and *nod* gene-lacking *Aeschynomene* bradyrhizobia.

## Materials and Methods

### Bacterial strains and DNA preparation

*Bradyrhizobium* sp. DOA9 was cultured for 4 d at 28°C in arabinose–gluconate medium [9]. Genomic DNA was prepared as described by Wilson [10].

### Sequencing and annotation

The genome sequence of DOA9 was determined by 454 pyrosequencing analysis using a GS FLX Titanium system (Roche Diagnostics Co., Indianapolis, IN, USA). Genomic DNA (5 μg) was sheared using nebulization to obtain fragments ranging from 300 to 800 bp. Template DNA was prepared according to the supplier’s protocol. The pyrosequencing data were assembled using the MIRA assembler ver. 3 [11], and were curated by comparison with the Newbler ver. 2.8 (Roche Diagnostics Co., Indianapolis, IN, USA) assembly or with some close reference strains. Gap closing and resequencing of low-quality regions of the assembled data were performed by PCR and Sanger sequencing. Regions encoding structural RNAs, rRNAs, tRNAs, tmRNAs, noncoding RNAs, and proteins were predicted using the Genaris Annotation System (Genaris, Inc., Kanagawa, Japan).

### Genome comparisons and ortholog analysis

A circular genome map showing the GC skew and the GC content was created using the CGview server [12] with default parameters. Similarity was compared between DOA9 and other bacteria using GenomeMatcher [13]. Putative orthologous genes among *Bradyrhizobium* strains DOA9, USDA110, and ORS278 were identified using bidirectional BLASTN comparisons with an e-value cut-off of 10^−20^. Orthologous relationships were depicted in a Venn diagram. Phylogenetic analysis was performed by comparing the sequences aligned using the CLUSTALW program [14]. Neighbor-joining trees were constructed using MEGA version 5.02 [15] and 1,000 bootstrap replicates were used to generate a consensus tree.

### Pulse-field gel electrophoresis

DNA plugs for pulse-field gel electrophoresis were prepared using CHEF Genomic DNA Plug Kits (Bio-Rad Laboratories Inc., Hercules, CA, USA) as follows. DOA9 was cultured for 6 d at 28°C in peptone salts yeast extract medium [16]. Bacterial cells were collected by centrifugation, washed twice with 0.85% NaCl, and resuspended with 0.85% NaCl to an OD_600_ of 5. The cell suspension (0.5 mL) was centrifuged at 8,000 × *g* for 5 min at 10°C, then resuspended with 0.5 mL of cell suspension buffer and mixed thoroughly with 0.5 mL of 2% CleanCut agarose (Bio-Rad Laboratories Inc., Hercules, CA, USA). The mixture was transferred to plug molds and solidified at 4°C for 30 min. The solidified agarose plugs were treated with lysozyme (1 mg/mL) at 37°C for 4 h and with proteinase K (30 U/mL) at 50°C overnight. Fragments of 225–6,000 kb and 225–2,200 kb were separated on 0.8% certified megabase agarose (Bio-Rad Laboratories Inc., Hercules, CA, USA) in TAE buffer or 1% certified megabase agarose in 0.5xTBE buffer, respectively. Contour-clamped homogeneous electric field (CHEF) electrophoresis was conducted at 14°C in a temperature-controlled cooling unit using the autoalgorithm mode in the CHEF Mapper gel electrophoresis system (Bio-Rad Laboratories Inc., Hercules, CA, USA). The gel was stained with 0.5 mg mL^−1^ ethidium bromide for 1 h and then destained in H_2_O for 1 h.

## Results

### Phylogeny of *Bradyrhizobium* sp. DOA9

**Fig 1:**
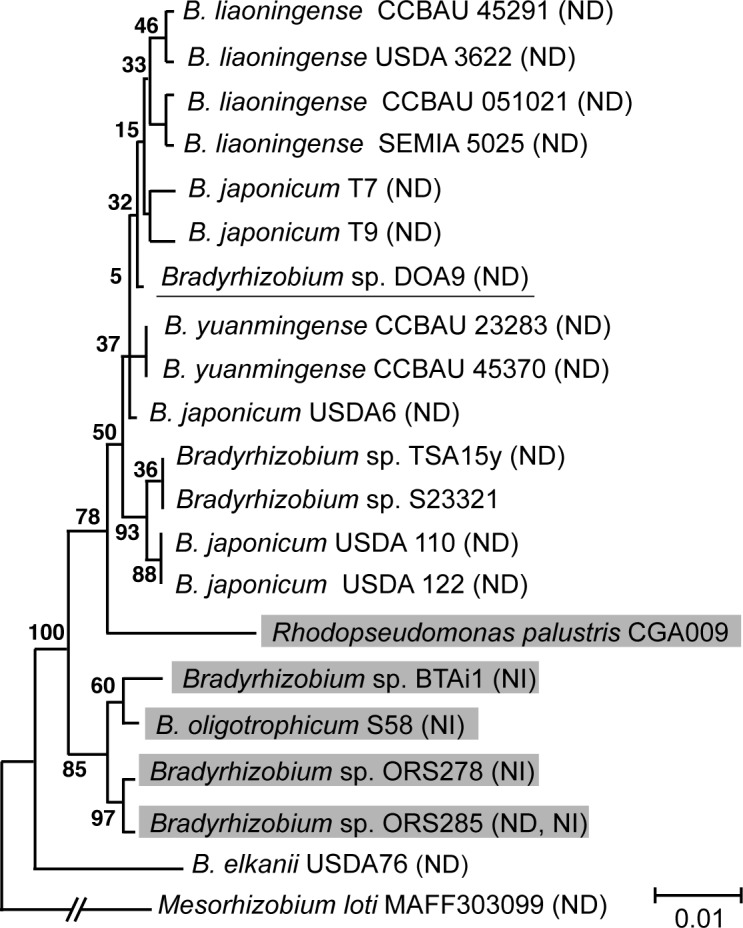
Phylogenetic relationships of *Bradyrhizobium* sp. DOA9 and related bacteria based on 16S rRNA gene sequences. Bootstrap values are expressed as percentages of 1,000 replications. Evolutionary distances were computed using the Kimura two-parameter method. The bar represents one estimated substitution per 100-nucleotide positions. Strains capable of Nod factor-dependent and -independent nodulation are marked with (ND) and (NI), respectively. Photosynthetic strains are highlighted in gray.

To examine the relationships between *Bradyrhizobium* sp. DOA9 and other members of the Bradyrhizobiaceae, phylogenetic trees were constructed based on 16S rRNA and internal transcribed spacer sequences (Fig. 1 and S1 Fig.). Using both approaches, DOA9 sequences were clustered within a group of **nonphotosyntetic** *nod* gene-containing bradyrhizobia, including *B*. *japonicum*, *B*. *liaoningense*, and *B*. *yuamingense* and were separated from those of photosynthetic *nod* gene-lacking strains, such as *Bradyrhizobium* spp. ORS278 and BTAi1. These results suggest that the DOA9 genome resembles more closely those of nonphotosynthetic *nod* gene-containing soybean bradyrhizobia than those of photosynthetic *nod* gene-lacking bradyrhizobia.

### Sequencing

The nucleotide sequence of the DOA9 genome was deduced by assembling 454 pyrosequencing data and Sanger sequencing data. In total, 1,853,745 reads from 454 pyrosequencing data were assembled using the MIRA assembler (version 4.0), and 591 contigs were generated. After curation of the MIRA software assembly by comparison with the Newbler software assembly or with the genomes of close reference strains, 48 contigs remained. Gap closing and resequencing of low-quality regions in the assembled data were performed by PCR and Sanger sequencing.

**Fig 2:**
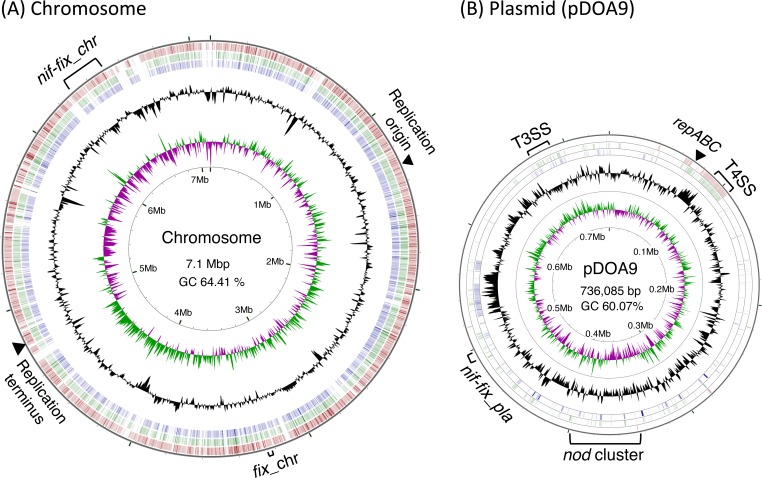
The genome structure of *Bradyrhizobium* sp. DOA9. (A) Circular representation of the chromosome of *Bradyrhizobium* sp. DOA9. The outermost, second, and third circles represent BLASTN comparisons with *B*. *japonicum* USDA110, *Bradyrhizobium* sp. ORS278, and *Bradyrhizobium* sp. ORS28, respectively (e-value < 10^−10^). The innermost and second-innermost circles show the GC skew and the GC content, respectively. The GC content circle shows the deviation from the average GC content of the entire sequence (higher than average GC content is represented in green, and lower than average content is represented in purple). The markings inside the innermost circle represent genome positions (in Mb). The positions of the putative replication origin, putative replication terminus, and nitrogen-fixation genes are shown outside of the outermost circle. (B) Circular representation of the plasmid (pDOA9) of DOA9. The outermost, second, and third circles represent BLASTN comparisons with the plasmid pBBta01 of *Bradyrhizobium* sp. BTAi1, the draft genome of *Bradyrhizobium elkanii* 587 (GenBank accession number AJJK00000000), and *B*. *japonicum* USDA110, respectively (e-value < 10^−10^). The innermost and second-innermost circles show the GC skew and the GC content, respectively. The GC content circle shows the deviation from the average GC content of the entire sequence (higher than average GC content in represented in green, and lower than average is represented in purple). The markings inside the innermost circle represent genome positions (in Mb). The positions of the *repABC* operon, T3/T4SS, *nod* genes, *nif-fix* gene clusters, and *hup* cluster are shown outside of the outermost circle.

The final draft genome consisted of two circular scaffolds of about 7.1 Mbp and 0.7 Mbp (Fig. 2). The 7.1-Mb scaffold comprised five contigs and was considered to be the main chromosome of DOA9 (Fig. 2A). Due to the presence of small but highly repetitive elements, five gaps of 1–6 kb in the scaffold could not be closed. The smaller scaffold consisted of one contig of 736,085 bp (Fig. 2B) and was considered to be the plasmid.

**Fig 3:**
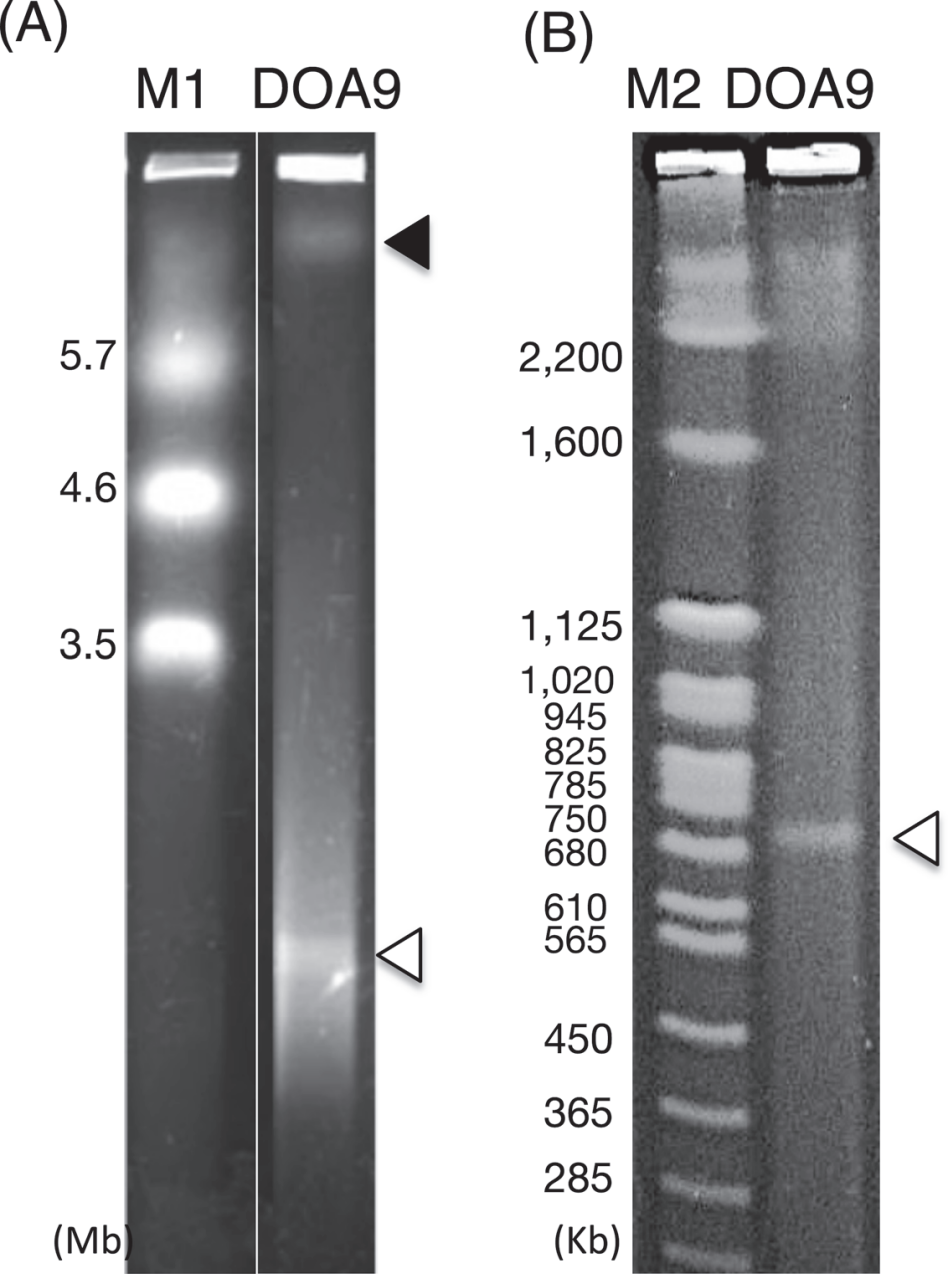
Pulse-field gel electrophoresis of *Bradyrhizobium* sp. DOA9 genomic DNA. DOA9 cells were digested in 1% pulse field grade (PFG) agarose plugs with proteinase K, as described in the Materials and Methods, and run 0.8% certified megabase agarose in TAE buffer to separate fragments of 225–6,000 kb (A), or 1% certified megabase agarose in 0.5 x TBE buffer (B) to separate fragments of 225–2,200 kb, respectively. Closed arrowheads and open arrowheads indicate the putative chromosome and the plasmid, respectively. Lane M1: PFGE marker, 3.5–5.7 Mb, *Saccharomyces pombe* chromosomal DNA. Lane M2: low-range (2.03–194 kb) PFG marker DNA ladder. DOA9: DOA9 genomic DNA.

In order to confirm the replicon structure of DOA9, genomic DNA was analyzed by pulse-field gel electrophoresis. A larger fragment (more than 5 Mbp) and a smaller fragment (between 680 and 750 kb) were detected (Fig. 3), similar in size to the larger scaffold (7.1 Mb) and the smaller scaffold (736,085 bp) of the sequencing results, respectively. The canonical *repABC* operon, which is required for the replication of bacterial plasmids, was found in the smaller scaffold (BDOA9_0200660 to BDOA9_0200680, see below). Therefore, we concluded that the smaller scaffold was the plasmid of DOA9 and designated it as pDOA9. The sequences of the chromosome and plasmid pDOA9 are available in the DDBJ/GenBank/EMBL database (accession numbers DF820425 and DF820426, respectively).

### General genome description

The average GC contents of the chromosome and the plasmid were 64.41% and 60.07%, respectively (Table 1). A GC skew analysis was performed to predict the locations of the putative replication origin and terminus of the chromosome. Two shifts of the GC skew were observed in the chromosome at coordinates 1.3 Mb and 4.86 Mb, respectively (Fig. 2A, innermost circle). The putative replication origin determined by comparison with the genome of *B*. *japonicum* USDA110 was found at 1.3 Mb. In *B*. *japonicum* USDA110, the putative replication origin was positioned between two conserved hypothetical proteins bll0636 and blr0637 [4]. The orthologs of these two genes, BDOA9_0112090 and BDOA9_0112080, were found at coordinates 1,313,579 to 1,313,070 and 1,312,684 to 1,311,839, respectively. The *dif* sequence, which is required to convert a dimer chromosome to monomers after aberrant DNA duplication, was found at coordinates 4,862,982 to 4,863,009 (Fig. 2A). The DNA replication may terminate in this region.

**Table 1:** General genome features of DOA9 and related bradyrhizobiaceae

	*B*. *japonicum*		*Bradyrhizobium* sp.			
Strain	USDA110	USDA6	DOA9^a^	ORS285	ORS278	BTAi1
Genome size (bp)	9,105,828	9,207,384	7,850,677^b^ (736,085)	7,632,258	7,456,587	8,493,515
G + C content (%)	64.06	63.67	64.41 (60.07)	65.23	65.51	64.80
tRNA coding genes	50	51	50 (1)	51	51	51
rRNA genes	3	6	3 (0)	4	6	6
Genes	8,374	8,882	7273 (676)	6,848	6,748	7,723
Photosynthetic	-	-	-	-	+	+
CI group	1	1	1	2	3	3
Nod-dependent (ND) or Nod-independent (NI)	ND	ND	ND	ND and NI	NI	ND
Original host plant	Soybean	Soybean	Aeschynomene	Aeschynomene	Aeschynomene	Aeschynomene

^a^ The values of plasmid is shown in parentheses.

^b^ The estimated values with gaps.

DNA dot plot analysis was performed to compare the genome structures of Bradyrhizobiaceae. The genome of DOA9 showed extensive similarity with the chromosome of the nonphotosynthetic *B*. *japonicum* spp. USDA6 and USDA110 and the photosynthetic non-nodulating *Bradyrhizobium* sp. S23321 (S2 Fig.). Conversely, the chromosome of DOA9 showed low similarity with those of the photosynthetic strains, including *Bradyrhizobium* spp. BTAi1, ORS278, and S58 (S2 Fig.). These results are in agreement with the phylogenetic analysis based on 16S rRNA gene sequences (Fig. 1).

### RNA- and protein-encoding genes

The DOA9 genome contains one copy of the rRNA gene cluster at coordinates 1,939,820 to 1,939,935 of the chromosome. In total, 50 tRNA genes, which correspond to all 20 of the standard amino acids, were scattered throughout the main chromosome. A total of 7,326 (chromosome: 6,650; plasmid: 676) opening reading frames were predicted and annotated.

**Fig 4:**
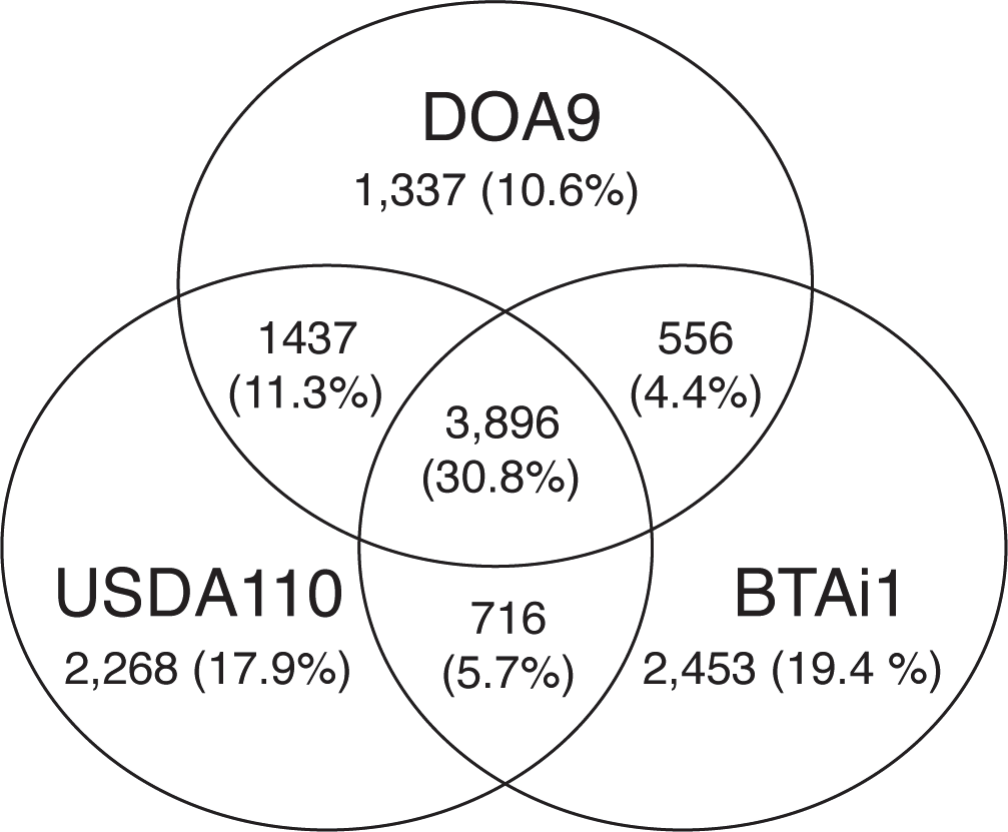
Comparative genomic analysis among *Bradyrhizobium* sp. DOA9, *B*. *japonicum* USDA110, and *Bradyrhizobium* sp. BTAi1. Each genome is represented by a circle, and the numbers of shared and unique genes are shown by the overlapping and nonoverlapping regions. The proportion of total genes represented by each area of the diagram is shown in parentheses. The total number of genes in each genome is shown in square brackets.

A BiBlast comparison was conducted among bradyrhizobial strains DOA9, USDA110, and BTAi1 (Fig. 4). Overall, 3,896 genes (30.8%) are conserved among all three strains (Fig. 4) representing 53.6% (3,896/7,273) of the total number of DOA9 genes. The number of genes unique to DOA9 (1,337 genes; 10.6%) is lower than that of both *B*. *japonicum* sp. USDA110 (2,268 genes; 17.9%) and *Bradyrhizobium* sp. BTAi1 (2,453 genes; 19.4%). DOA9 and *B*. *japonicum* sp. USDA110 share 1,437 genes (11.3%) that were not found in *Bradyrhizobium* sp. BTAi1—a markedly higher number than the 556 genes (4.4%) shared by DOA9 and BTAi1. These results indicate that the DOA9 genome is similar to the genome of *B*. *japonicum* USDA110 in terms of gene content.

### Properties of pDOA9

**Fig 5:**
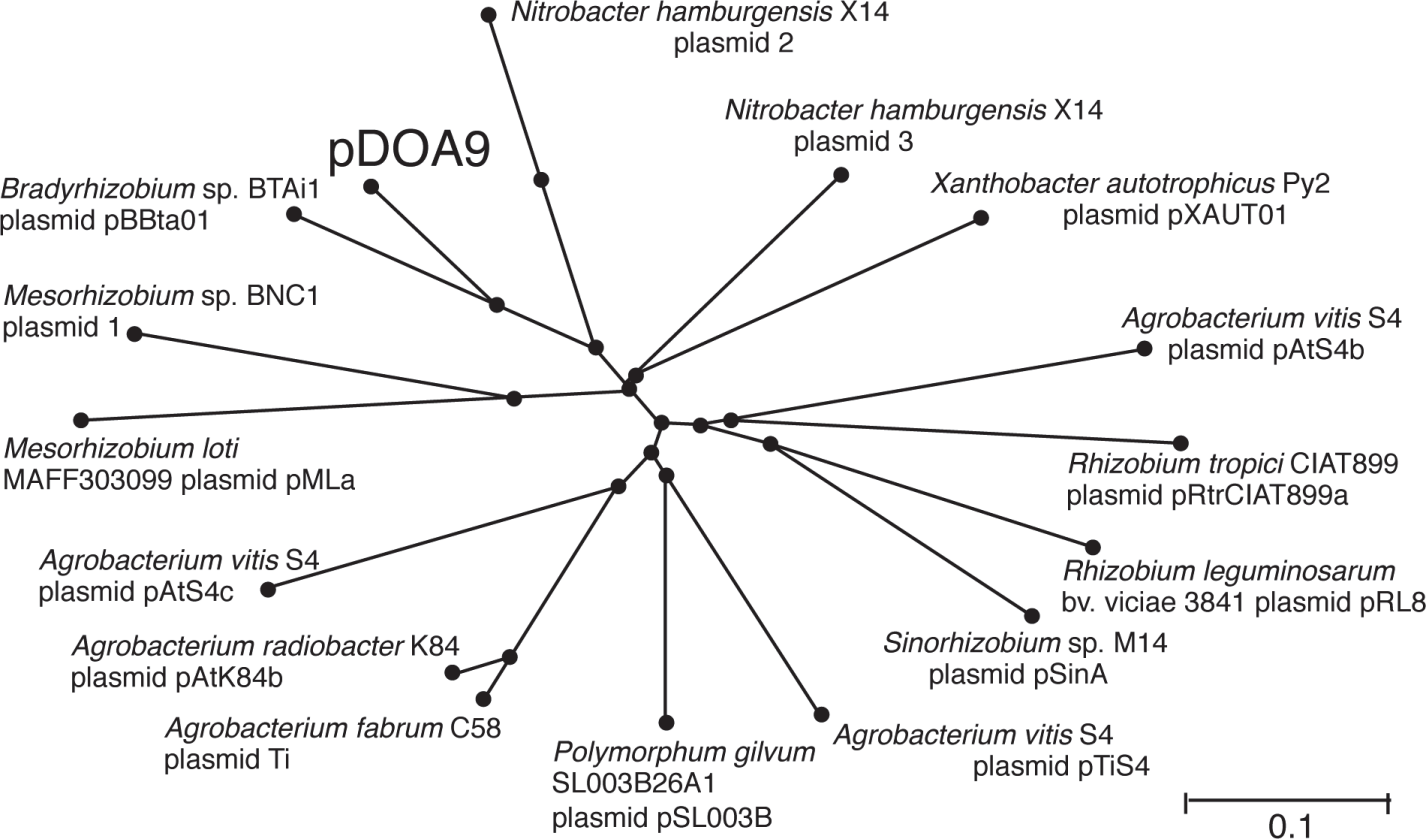
Phylogenetic relationships of pDOA9 and related plasmids. Bootstrap values are expressed as percentages of 1,000 replications. Evolutionary distances were computed using the Kimura two-parameter method. The bar represents one estimated substitution per 100-nucleotide positions.

The BLASTN comparison indicated that pDOA9 showed similarity to the plasmids of *Rhizobium* and *Agrobacterium*. The dendrogram based on genomic dissimilarity revealed that pDOA9 is grouped with pBBTa01of *Bradyrhizobium* sp. BTAi1 (Fig. 5). There was no evidence of extensive synteny between pDOA9 and pBBTa01 or other symbiotic plasmids, except in gene regions with symbiosis-related functions such as nodulation, nitrogen fixation, type III/IV secretion systems, and hydrogenase (Fig. 2B). The GC content of pDOA9 (60.07%) was lower than that of the chromosome (64.95%); this characteristic is similar to *Bradyrhizobium* sp. BTAi1, where the GC content is 60.69% for pBBTa01 and 64.92% for the chromosome.

### Nodulation genes

**Fig 6:**
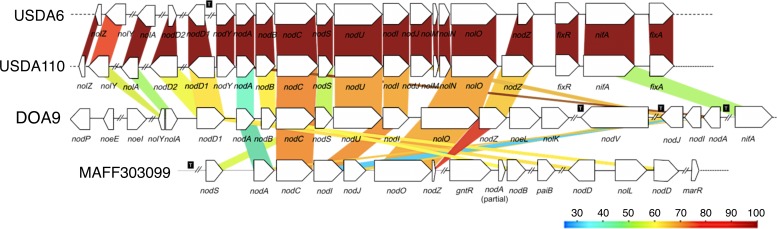
Comparison of nodulation gene clusters in *Bradyrhizobium* sp. DOA9 and related bacteria. Double slash marks represent DNA regions that are not shown. Colored strips represent the conserved gene regions between the compared strains, and the color indicates the percentage similarity, as detailed in the key. T: region where the transposase genes were located.

Nodulation genes encode proteins involved in the biosynthesis and transport of NFs, which induce nodule organogenesis in most rhizobia. A large *nod* gene cluster was found on pDOA9 (Fig. 2B). The organization and repertoire of *nod* genes are similar to those of *B*. *japonicum*, although losses, insertions, and inversions were found (Fig. 6 and Table 2). For example, *B*. *japonicum* USDA110 possesses *nodYABCSUIJnolMNO* arranged in a single operon, whereas DOA9 lacks *nodY*, *nodJ*, *nolM*, and *nolN*. In addition, *B*. *japonicum* has two *nodD* genes (*nodD1* and *nodD2*) within the cluster, whereas DOA9 has only one (*nodD1*, locus tag: BDOA9_0203500) in the cluster and the other (*nodD2*, BDOA9_0205810) located 0.27 Mb away. Furthermore, *nodQ*, *nodP*, *noeE*, *noeL*, and *nolK* (BDOA9_0203370–0203390, 0203580–0203590) are incorporated into the *nod* gene cluster of DOA9 but are located far from the cluster in *B*. *japonicum*. Unlike *B*. *japonicum* USDA110, DOA9 possesses two copies of *nodA* (*nodA1*, BDOA9_0206740 and *nodA2*, BDOA9_0203720), one in the *nodABCSUI* operon and the other in the *nodA2IJ* operon. *nodA1* shares only 32–36% identity with the copies in other *Bradyrhizobium* strains, whereas *nodA2* shares 63–69% identity (Table 2). *nodB* and *nodC* share more than 60% identity with the corresponding genes in *B*. *japonicum* (Table 2). Phylogenetic analyses of the common nodulation genes *nodA*, *nodB* and *nodC* showed that those of DOA9 were placed on unclassified branches separated from the known *nod* gene-containing rhizobia (S3 Fig.). Although most of the *nod* genes involved in NF biosynthesis, such as *nodABC*, are located on pDOA9, some are also found on the chromosome (Table 2): *nolG*, *nodV*, *nodW*, *nodQ*, *nodN*, and *nodT*. Eight *nod* box consensus sequences [17], where NodD (the transcriptional activator that responds to flavonoids) binds to the DNA, were identified in pDOA9 (S4 Fig.). Most of the *nod* boxes preced genes involved in Nod factor biosynthesis and regulation, as in other rhizobia. No *nod* boxes were identified in the chromosome using our search criteria.

**Table 2:** Nodulation genes detected in the genome of *Bradyrhizobium* sp. DOA9 and higher BLASTP similarity with other microorganisms.

Gene	Locus tag	Percentage of identity in BLASTP
Chromosome		
*nolG*	BDOA9_0103430	*Bradyrhizobium* sp. BTAi1 (91%), *B*. *japonicum* USDA110 (90%), *Bradyrhizobium* sp. ORS278 (76%), *R*. *leguminosarum* bv. *viciae* 3841 (74%)
*nolG*	BDOA9_0108870	*B*. *japonicum* USDA110 (95%), *Bradyrhizobium* sp. ORS278 (83%), *Bradyrhizobium* sp. BTAi1 (83%), *A*. *caulinodans* ORS571 (56%)
*nodV*	BDOA9_0120640	*B*. *japonicum* WSM2793 (40%), *Methylobacterium* sp. 10 (31%), *Rhizobium* sp. PDO1–076 (31%)
*nodQ*	BDOA9_0121760	*B*. *japonicum* USDA110 (96%), *Bradyrhizobium* sp. ORS278 (80%), *Bradyrhizobium* sp. BTAi1 (80%), *A*. *caulinodans* ORS571 (60%)
*nodW*	BDOA9_0136710	*B*. *japonicum* USDA110 (76%), *S*. *meliloti* 1021 (58%)
*nodW*	BDOA9_0136720	*B*. *japonicum* USDA110 (66%), *R*. *leguminosarum* bv. viciae 3841 (40%)
*nodV*	BDOA9_0136850	*B*. *japonicum* USDA110 (67%), *S*. *meliloti* 1021 (35%)
*nodN*	BDOA9_0136940	*B*. *japonicum* USDA110 (92%), *C*. *taiwanensis* LMG 19424 (43%)
*nodN*	BDOA9_0139450	*B*. *japonicum* USDA110 (96%), *Bradyrhizobium* sp. BTAi1 (83%), *Bradyrhizobium* sp. ORS278 (83%), *M*. *loti* MAFF303099 (46%)
*nolG*	BDOA9_0140510	*B*. *japonicum* USDA110 (97%), *Bradyrhizobium* sp. BTAi1 (90%), *Bradyrhizobium* sp. ORS278 (90%), *A*. *caulinodans* ORS571 (76%)
*nodV*	BDOA9_0147240	*B*. *japonicum* USDA110 (61%), *R*. *leguminosarum* bv. *viciae* 3841 (32%)
*nodW*	BDOA9_0147250	*B*. *japonicum* USDA110 (78%), *A*. *caulinodans* ORS571 (62%)
*nodW*	BDOA9_0147260	*B*. *japonicum* USDA110 (67%), *A*. *caulinodans* ORS571 (45%)
*nodV*	BDOA9_0151030	*B*. *japonicum* USDA110 (88%), *A*. *caulinodans* ORS571 (34%)
*nodW*	BDOA9_0151040	*B*. *japonicum* USDA110 (94%), *A*. *caulinodans* ORS571 (67%)
*nodW*	BDOA9_0151050	*B*. *japonicum* USDA110 (89%), *A*. *caulinodans* ORS571 (44%)
*nolG*	BDOA9_0151210	*Bradyrhizobium* sp. S23321 (98%), *B*. *japonicum* USDA110 (96%)
*nodW*	BDOA9_0151770	*B*. *japonicum* USDA110 (89%), *R*. *etli* CFN42 (50%), *C*. *taiwanensis* LMG 19424 (47%)
*nodV*	BDOA9_0153480	*B*. *japonicum* USDA110 (85%), *Bradyrhizobium* sp. BTAi1 (62%), *Bradyrhizobium* sp. ORS278 (61%)
*nolG*	BDOA9_0156320	*B*. *japonicum* USDA110 (91%), *Bradyrhizobium* sp. BTAi1 (87%), *Bradyrhizobium* sp. ORS278 (86%)
*nolO*	BDOA9_0158070	*B*. *japonicum* USDA6 (97%), *R*. *etli* CFN42 (35%), *M*. *loti* MAFF303099 (35%)
*nodT*	BDOA9_0162260	*Bradyrhizobium* sp. ORS278 (56%), *Bradyrhizobium* sp. BTAi1 (55%), *S*. *fredii* NGR234 (54%)
*nolG*	BDOA9_0165280	*B*. *japonicum* USDA110 (97%), *Bradyrhizobium* sp. BTAi1 (86%), *Azospirillum* sp. B510 (52%)
Plasmid (pDOA9)		
*nodW*	BDOA9_0203300	*B*. *japonicum* USDA110 (65%), *Azoarcus* sp. BH72 (42%)
*nodW*	BDOA9_0203310	*B*. *japonicum* USDA110 (67%), *Azoarcus* sp. BH72 (57%)
*nodQ*	BDOA9_0203370	*Bradyrhizobium* sp. ORS278 (53%), *Mesorhizobium loti* MAFF303099 (48%), *Sinorhizobium fredii* NGR234 (48%)
*nodP*	BDOA9_0203380	*Bradyrhizobium* sp. ORS278 (73%), *Azospirillum* sp. B510 (71%), *Bradyrhizobium* sp. BTAi1 (70%)
*noeE*	BDOA9_0203390	*S*. *fredii* NGR234 (55%)
*nodW*	BDOA9_0203420	*Mesorhizobium* sp. WSM4349 (33%), *S*. *terangae* WSM1721 (31%)
*noeI*	BDOA9_0203430	*B*. *japonicum* USDA110 (73%), *S*. *fredii* NGR234 (71%)
*nolA*	BDOA9_0203490	*Bradyrhizobium* sp. NC92 (62%), *Mesorhizobium* sp. WSM4349 (60%), *Bradyrhizobium* sp. ORS 285 (47%)
*nodD1*	BDOA9_0203500	*Rhizobium etli* CFN42 (63%), *B*. *japonicum* USDA110 (62%), *S*. *meliloti* 1021 (61%), *S*. *fredii* NGR234 (61%)
*nodA1*	BDOA9_0206740	*Bradyrhizobium* sp. WSM1417 (36%), *Rhizobium etli* CFN42 (34%), *B*. *japonicum* USDA110 (32%)
*nodB*	BDOA9_0203510	*S*. *fredii* NGR234 (65%), *S*. *fredii* HH103 (65%), *B*. *japonicum* USDA110 (62%), *Azorhizobium caulinodans* ORS571 (42%), *Azospirillum* sp. B510 (35%)
*nodC*	BDOA9_0203520	*B*. *japonicum* USDA110 (69%), *M*. *loti* R7A (68%), *R*. *etli* CFN42 (66%)
*nodS*	BDOA9_0203530	*S*. *fredii* NGR234 (59%), *R*. *etli* CFN42 (55%), *M*. *loti* MAFF303099 (55%), *B*. *japonicum* USDA6 (52%)
*nodU*	BDOA9_0203540	*B*. *japonicum* USDA110 (69%), *Cupriavidus taiwanensis* LMG 19424 (64%), *A*. *caulinodans* ORS571 (54%)
*nodI*	BDOA9_0203550	*M*. *loti* MAFF303099 (70%), *S*. *meliloti* 1021 (70%), *R*. *etli* CFN42 (68%)
*nolO*	BDOA9_0203560	*B*. *japonicum* USDA110 (68%), *M*. *loti* MAFF303099 (67%), *Rhizobium* sp. NGR234 (67%), *R*. *etli* CFN42 (65%)
*nodZ*	BDOA9_0203570	*R*. *etli* CFN42 (66%), *M*. *loti* MAFF303099 (66%), *S*. *fredii* NGR234 (64%)
*noeL*	BDOA9_0203580	*B*. *japonicum* USDA110 (91%), *Bradyrhizobium* sp. BTAi1 (84%)
*nolK*	BDOA9_0203590	*B*. *japonicum* USDA110 (89%), *Bradyrhizobium* sp. BTAi1 (80%), *Bradyrhizobium* sp. ORS278 (80%)
*nodV*	BDOA9_0203630	*B*. *japonicum* USDA110 (50%)
*nodJ*	BDOA9_0203700	*R*. *etli* CFN42 (66%), *M*. *loti* MAFF303099 (64%), *S*. *fredii* NGR234 (64%), *R*. *leguminosarum* bv. *viciae* 3841 (64%)
*nodI*	BDOA9_0203710	*B*. *japonicum* USDA110 (47%), *M*. *loti* MAFF303099 (38%)
*nodA2*	BDOA9_0203720	*B*. *japonicum* USDA110 (71%), *S*. *fredii* NGR234 (70%), *M*. *loti* R7A (69%), *C*. *taiwanensis* LMG 19424 (69%)
*nolY*	BDOA9_0203790	*B*. *japonicum* USDA110 (42%), *S*. *fredii* NGR234 (33%)
*nodV*	BDOA9_0205070	*B*. *japonicum* USDA110 (85%), *Bradyrhizobium* sp. BTAi1 (79%), *Bradyrhizobium* sp. ORS278 (79%)
*nodD2*	BDOA9_0205810	*B*. *japonicum* USDA110 (69%), *S*. *meliloti* 1021 (66%), *R*. *leguminosarum* bv. *viciae* 3841 (63%)

### Nitrogen fixation genes

In DOA9 a single cluster of *fix* genes (*fix*_*chr*) was found on the chromosome and a *nif-fix* cluster was found on both the chromosome (*nif-fix_chr*) and the plasmid (*nif-fix*_*pla*) (Fig. 2 and S5 Fig.). The *fix*_*chr* cluster includ *fixK-fixLJ*, which encodes a factor involved in the regulation of symbiotic nitrogen fixation [18], *fixNOQP*, which encodes a microaerobically induced cytochrome oxidase complex [19], and *fixGHIS*, which encodes a membrane-bound complex including a cation pump involved in nitrogen fixation [19]. The comparative analysis showed that the organization of the *fix*_*chr* cluster is conserved among bradyrhizobial strains (S5 Fig.).

Comparative analysis showed that the organization of the *nif-fix*_*chr* cluster is almost identical to that in photosynthetic bradyrhizobial strains, including *Aeschynomene* symbionts (*Bradyrhizobium* spp. ORS278, BTAi1, S58, and ORS285) and the non-nodulating strain *Bradyrhizobium* sp. S23321. An exception was found for the *nifH* gene: photosynthetic bradyrhizobial strains possess two copies of *nifH*, which are located in the *nifHQ* and *nifHDK* operons; by contrast, DOA9 possesses only the *nifHQ* operon, and another copy of *nifH* might have been deleted from the *nifHDK* operon (S6 Fig.). Notably, a copy of *nifV*, which encodes the homocitrate synthase [20], was found in the cluster, similar to the photosynthetic strains *Bradyrhizobium* spp. ORS285, S58, BTAi1, ORS278, and S23321.

Another *nif-fix* cluster (*nif-fix_pla*) was also found in the plasmid of DOA9 (Fig. 2B and S6 Fig.). The *nif-fix_pla* is more divergent than the *nif-fix_chr* especially *nifQ* (BDOA9_0204570), which is involved in the incorporation of molybdenum into nitrogenase, and *nifW* (BDOA9_0204580), which is a nitrogenase stabilizing and protective protein. The *nifH* gene in the chromosome (BDOA9_0160870) and the plasmid (BDOA9_0204560) show 96% identity. The phylogenetic analysis showed that *nifH* of DOA9 is placed on a separate branch, distinct from those of nonphotosynthetic species, such as *B*. *japonicum*, *B*. *elkanii*, and *B*. *yuanmingense* (S7 Fig.). *nifH* on the chromosome of DOA9 shares higher similarity with the same gene of photosynthetic strains than that of nonphotosynthetic strains.

### Gene clusters of type III /IV secretion systems

A cluster of genes encoding the type III secretion system was found in pDAO9 spanning a 53-kb region. The organization of genes encoding the type III secretion apparatus (*rhcC*, *rhcJ*, *rhcN*, and *rhcQRSTU*) is well conserved among bradyrhizobial strains (S2 Table and S8 Fig.). The organization of the *tts* gene cluster of DOA9 is similar to that of *B*. *elkanii* USDA61, although losses, insertions, and inversions of some genes are present. Phylogenetic analysis using the conserved type III apparatus *rhcTVRUC* genes showed that the *tts* genes of DOA9 are grouped with those of other *Bradyrhizobium* strains (S8 Fig.). The DOA9 genome contains 10 *tts* boxes, which are conserved motifs located in the promoter region of genes encoding structural components and secrete proteins of the rhizobial *tts* cluster [21] (S9 Fig.). Most of the *tts* boxes precede genes that encode nodulation outer proteins (*nopB* and *nopX*) or structural components of the type III secretion machinery (*y4yQ* and *rhcT*). *tts* boxes were also identified in the upstream regions of the genes encoding ParA-like ATPase, heat shock protein, cupin, and hypothetical proteins. Homologues of these genes have not been reported so far as type III secreted proteins or related components.

The plasmid pDOA9 also contains genes encoding a VirB/D4 type IV secretion system (T4SS). The *vir* cluster includes *virB1* to *virB11*, which encode the transmembrane complex and pili required for transfer proteins, and *virD4*, which encodes a protein that links the transferred substrates to the transfer apparatus (Ding *et al*. 2003). The *vir* genes of DOA9 are similar to that of BTAi1 (S10 Fig. and S3 Table). The phylogenetic analysis of two representative genes, *virB2* and *virB9*, supports this finding (S10 Fig.).

## Discussion

*Bradyrhizobium* sp. DOA9 is of particular biological interest because it possesses a broader host range and divergent *nod* genes compared with other bradyrhizobia [7]. The most prominent characteristic of the DOA9 genome is the presence of symbiosis-related genes on the plasmid rather than on the chromosome. A similar trait is seen in fast-growing rhizobia, whose symbiosis-related genes are often clustered on a large plasmid, such as pNGR234a (536,165 bp) of *Sinorhizobium fredii* NGR234 [22], p42d of *Rhizobium etli* CFN42 (371,254 bp) [23], and pRL10 of *Rhizobium leguminosarum* biovar 3841 (488,135 bp) [24]. Although plasmids were found in *Bradyrhizobium* sp. BTAi1 and some soybean bradyrhizobia, they did not contain any *nod* genes [25]. In *nod* gene-carrying bradyrhizobial strains such as *B*. *japonicum* USDA110, the symbiosis-related genes are clustered in regions of the chromosome with a low GC content, known as symbiosis islands [4,5]. For instance, in *B*. *japonicum* USDA110 the symbiosis island is a 680-kb region with a relatively low GC content (59.4%) compared with that of the entire genome (64.1%). Notably, in DOA9 the GC content of pDOA9 (60.07%) was lower than that of the chromosome (64.41%). Symbiosis islands have been found to integrate genes of the recipients into the tRNA. Sullivan *et al*. reported [26] that the symbiotic island of *Mesorhizobium loti* was transferred to nonsymbiotic strains present in the soil. It is possible that pDOA9 can be transferred to other nonsymbiotic bradyrhizobia by conjugal transfer and integrated into their chromosomes through a process mediated by an integrase, as reported for many other elements, including the pathogenicity islands of pathogenic bacteria [27]. However, the details of the molecular mechanisms and host range for plasmid transfer remain to be elucidated; the latter is particularly interesting because the plasmid might enhance the fitness of bacteria.

The *nod* genes of DOA9 are highly divergent compared with those of other rhizobia in terms of both gene organization and homology. DOA9 is phylogenetically close to *B*. *japonicum* (Fig. 1), and their chromosomes share a high degree of similarity (S2 Fig.). However, their host legume specificities are different and DOA9 is unable to nodulate soybean [7]. In *B*. *japonicum*, the common *nodABC* genes are cotranscribed with *nodY* [28–30]. The *nod* gene cluster in DOA9 consists of the *nodABCSU* genes but lacks *nodY* and *nolMN* (Fig. 6). *nodY* was reported to be *Bradyrhizobium* specific and to be induced by soybean seed extract and selected isoflavones, primarily genistein and daidzein, but not by flavones [31]. This might account for the nodulation deficiency of DOA9 on soybean. It is possible that soybean bradyrhizobia acquired *nodY* during coevolution with soybean from *Aeschynomene* bradyrhizobia.

Unlike *B*. *japonicum*, DOA9 harbors additional host-specific genes, *nodQ*, *nodP*, and *noeE*, in the *nod* gene cluster (Fig. 6 and Table 2). These genes exhibit no similarity with those of *B*. *japonicum*, suggesting that DOA9 acquired them independently from other rhizobia. The roles of these genes in nodulation and host specificity remain to be elucidated. Moreover, DOA9 harbors two copies of *nodA* in the *nod* gene cluster: *nodA1*, which is divergent, is incorporated into the *nodA1BCSUI* operon, whereas *nodA2* is incorporated into the *nodA2IJ* operon. This structure is similar to the structure of *Rhizobium tropici*, which carries three copies of *nodA* on the symbiotic plasmid and whose *nodA2* and *nodA3* genes have no close homologues [32]. The NF acyl group attached by NodA might contribute to the determination of host range [33]. Additional and divergent *nodA* genes are likely to expand the diversity of NF acyl chains and might broaden the host range of the bacteria [33].

DOA9 harbors several copies of *nodVW* on both the chromosome and the plasmid: seven copies of *nodV* (five on the chromosome and two on the plasmid) and 10 copies of *nodW* (seven on the chromosome and three on the plasmid) (Table 2). NodVW is a member of the classical two-component regulatory family and is essential for the nodulation of cowpea, siratro, and mungbean [34]. In *B*. *japonicum*, NodVW is considered to recognize plant flavonoids and could therefore contribute to the nodulation of a broader range of host plants by increasing NF synthesis in combination with NodD [20,35]. Although most *nodVW* genes showed high similarity with those of *B*. *japonicum*, some—BDOA9_0120640, BDOA9_0203420, and BDOA9_0203630—exhibited low similarity (Table 2). These NodVW can recognize additional plant and environmental signals and hence activate symbiosis-related genes. Further study will be necessary to elucidate the involvement of *nodVW* genes in broad host specificity of DOA9.

The genome of DOA9 contains a full set of nitrogen-fixing genes (*nif-fix*) on the chromosome and an incomplete cluster of these genes on pDOA9. The *nif-fix* cluster on the chromosome (*nif-fix_chr*) is highly similar to that of photosynthetic bradyrhizobia as well as of the non-nodulating strain *Bradyrhizobium* sp. S23321. The *nif-fix* cluster on pDOA9 (*nif-fix_pla*) lacks most of the *nif* genes and those present are highly similar to those of photosynthetic bradyrhizobia. As in photosynthetic bradyrhizobia, *nif-fix*_*chr* cluster in DOA9 carries *nifV*, which encodes homocitrate synthase that is essential for nitrogenase activity in the free-living diazotrophs [20]. *nifV* is absent from most strains of rhizobia that perform nitrogen fixation only in symbiotic states, whereas it is presents in strains that also fix nitrogen in *ex planta* states such as *Azorhizobium caulinodans* [20], *Bradyrhizobium* spp. BTAi1 and ORS278 [36]. The presence of *nifV* in DOA9 suggests that DOA9 has retained the nitrogen-fixation system evolved from the ancestral photosynthetic bradyrhizobia and produces NifV to perform efficient nitrogen fixation during symbiosis.

Clusters of genes encoding elements of the type III secretion system (T3SS) were found in the genomes of various rhizobia including the nonphotosynthetic strains *B*. *japonicum* USDA6 and USDA110 and *B*. *elkanii* USDA61, and the photosynthetic strains *Bradyrhizobium* spp. ORS285; however, the cluster was not found in the genome of the most nod-independent bradyrhizobial strains BTAi1, ORS278, and S23321. The phylogenetic analysis using *rhcTVRUC* genes, which encode conserved proteins of the type III secretion apparatus, indicated that the genes encoding T3SS in DOA9 and other bradyrhizobial strains shared the same evolutionary origin (S8 Fig.). Among these, the components of the type III secretion apparatus of DOA9 showed high identities with those of the soybean symbionts *B*. *elkanii* USDA61 and *B*. *japonicum* USDA110 (S2 Table). The T3SS genes of these strains were expressed in the presence of the soybean flavonoid genistein, confirming that it functions in the infection of host roots. The induction of T3SS genes involves two regulators: NodD, which is activated by genistein; and TtsI, which is induced by the NodD protein. DOA9 possesses both of these regulators, and they show high identity with the soybean symbionts, despite the fact that DOA9 is unable to nodulate soybean. Expression analysis of T3SS genes and gene-deletion analysis could elucidate the regulation and symbiotic roles of T3SS in DOA9.

The VirB/D4 type IV secretion system (T4SS) has been identified in some rhizobia, including *M*. *loti* R7A[37] and *R*. *etli* CFN42[38] but is absent from bradyrhizobia with the exception of *Bradyrhizobium* sp. BTAi1[25]. *M*. *loti* R7A possesses a gene cluster (*vir*) encoding T4SS, similar to that of *Agrobacterium tumefaciens*. In *M*. *loti* R7A T4SS is transcriptionally regulated by a VirA/VirG two-component regulatory system [39]. In *M*. *loti* R7A the *virA* gene is preceded by a *nod* box, and T4SS is activated in a symbiosis-specific manner [39]. The DOA9 genome lack**s** homologues of *M*. *loti virA and virG*. Similarly, *virA* and *virG* homologues are absent from BTAi1, whereas *R*. *etli* CFN42 possess v***irA*** and v***irG*** that show 43% and 76% identities, respectively, with those of *M*. *loti* R7A. These findings suggest that the T4SS of *Bradyrhizobium* spp. DOA9 and BTAi1 share an origin that differs from that of *M*. *loti* R7A and other rhizobia.

In conclusion, the genome of DOA9 exhibits a mosaic structure with a unique repertoire of symbiotic genes with various origins. During adaptation to *A*. *americana* and other legumes, the ancestral strain of DOA9 acquired divergent nodulation genes and other symbiosis-related genes such as the type III and IV secretion systems. The present study also discovered the first symbiotic plasmid among *Bradyrhizobiaceae*. This plasmid will be a valuable tool for future studies on the genetics, physiology, and ecology of these species. For example, it will be interesting to test whether pDOA9 can transform non-symbiotic strains, such as S23321, into symbiotic strains or broaden the host range of BTAi1. Our efforts are now directed toward functional analysis of the divergent *nod* genes in pDOA9, to improve our understanding of the evolution of bradyrhizobia and of their host plants mediated by the symbiotic plasmid.

## Supporting Information

S1 Fig Phylogenetic relationships of *Bradyrhizobium* sp. DOA9 and related bacteria based on internal transcribe spaces sequences. Bootstrap values are expressed as percentages of 1,000 replications. Evolutionary distances were computed using the Kimura two-parameter method. The bar represents one estimated substitution per 100-nucleotide positions. Strains capable of Nod factor-dependent and -independent nodulation are marked with (ND) and (NI), respectively. Photosynthetic strains are highlighted in gray.
(TIF)

S2 Fig Comparison of chromosomal sequences of bradyrhizobial strains generated using the GenomeMatcher program computed by MUMmer. The positions on each chromosome of each bradyrhizobial strain are indicated on the x-axis and y-axis. The dot color indicates the percentage similarity, as indicated in the key.
(TIF)

S3 Fig Phylogenetic trees based on sequences of *nodA*, *nodB*, and *nodC* genes of DOA9 and other related rhizobia. Bootstrap values are expressed as percentages of 1,000 replications. The bar represents one estimated substitution per 100-nucleotide positions.
(TIF)

S4 Fig *nod* box sequences identified in *Bradyrhizobium* sp. DOA9. Nucleotides conserved in all cases are shown in bold uppercase letters. Numbers indicate the distance in base pairs between the *nod* box and the potential translational start site of the corresponding gene. In the consensus sequence capital letters are used for invariant nucleotides, and lowercase letters are used for nucleotides conserved in at least 50% of the sequences.
(TIF)

S5 Fig Comparative analysis of the *fix* cluster of *Bradyrhizobium* sp. DOA9 and related bacteria. Double slash marks represent DNA regions that are not shown. Colored strips represent the conserved gene regions between the compared strains, and the color indicates the percentage similarity, as indicated by the key.
(TIF)

S6 Fig Comparative analysis of the nitrogen-fixation gene clusters of *Bradyrhizobium* sp. DOA9 and related bacteria. Double slash marks represent DNA regions that are not shown. Colored strips represent the conserved gene regions between the compared strains, and the color indicates the percentage similarity, as indicated by the key. T: region where the transposase genes were located. N: region of nodulation genes. G: region of *groES-groEL* regulatory genes.
(TIF)

S7 Fig Phylogenetic trees based on sequences of *nifH* genes of DOA9 and other related bacteria. Bootstrap values are expressed as percentages of 1,000 replications. The bar represents one estimated substitution per 100-nucleotide positions.
(TIF)

S8 Fig Comparative analysis of T3SS genes clusters of *Bradyrhizobium* sp. DOA9 and related bacteria. Asterisks represent the strains harboring the T3SS cluster in the plasmid and each plasmid name is shown in parentheses. Double slash marks represent DNA regions that are not shown. Colored stripes represent the conserved gene regions between the compared strains, and the color indicates the percentage similarity, as indicated by the key. T: region where the transposase genes were located.
(TIF)

S9 Fig *tts* box sequences identified in *Bradyrhizobium* sp. DOA9. Nucleotides conserved in all cases are shown in bold uppercase letters. Numbers indicate the distance in base pairs between the *tts* box and the potential translational start site of the corresponding gene. In the consensus sequence capital letters are used for invariant nucleotides, and lowercase letters are used for nucleotides conserved in at least 50% of the sequences.
(TIF)

S10 Fig *vir* cluster of *Bradyrhizobium* sp. DOA9. (A) Comparative analysis of *vir* clusters of *Bradyrhizobium* sp. DOA9 and related bacteria. All the compared clusters were located on the symbiotic plasmid and each plasmid name is shown in parentheses. Double slash marks represent DNA regions that are not shown. Colored strips represent the conserved gene regions between the compared strains, and the color indicates the percentage similarity, as indicated by the key. (B) Phylogenetic trees based on a combination of the *virB2* and *virB9* sequences of DOA9 and other related rhizobia. Bootstrap values are expressed as percentages of 1,000 replications. The bar represents one estimated substitution per 100-nucleotide positions.
(TIF)

S1 Table Nitrogen fixation genes detected in the genome of *Bradyrhizobium* sp. DOA9 and higher BLASTP similarity with other microorganisms. (XLSX)

S2 Table Genes encoding the type III secretion system in the genome of *Bradyrhizobium* sp. DOA9 and higher BLASTP similarity with other microorganisms. (XLSX)

S3 Table Genes encoding the type IV secretion system in the genome of *Bradyrhizobium* sp. DOA9 and highter BLASTP similarity with other microorganisms. (XLSX)
