# Supplementary figures and images for: Genome Analysis of a Novel Bradyrhizobium sp. DOA9 Carrying a Symbiotic Plasmid
Source: PLoS One. 2015 Feb 24;10(2):e0117392. doi: 10.1371/journal.pone.0117392 (PMC4339197; doi:10.1371/journal.pone.0117392)

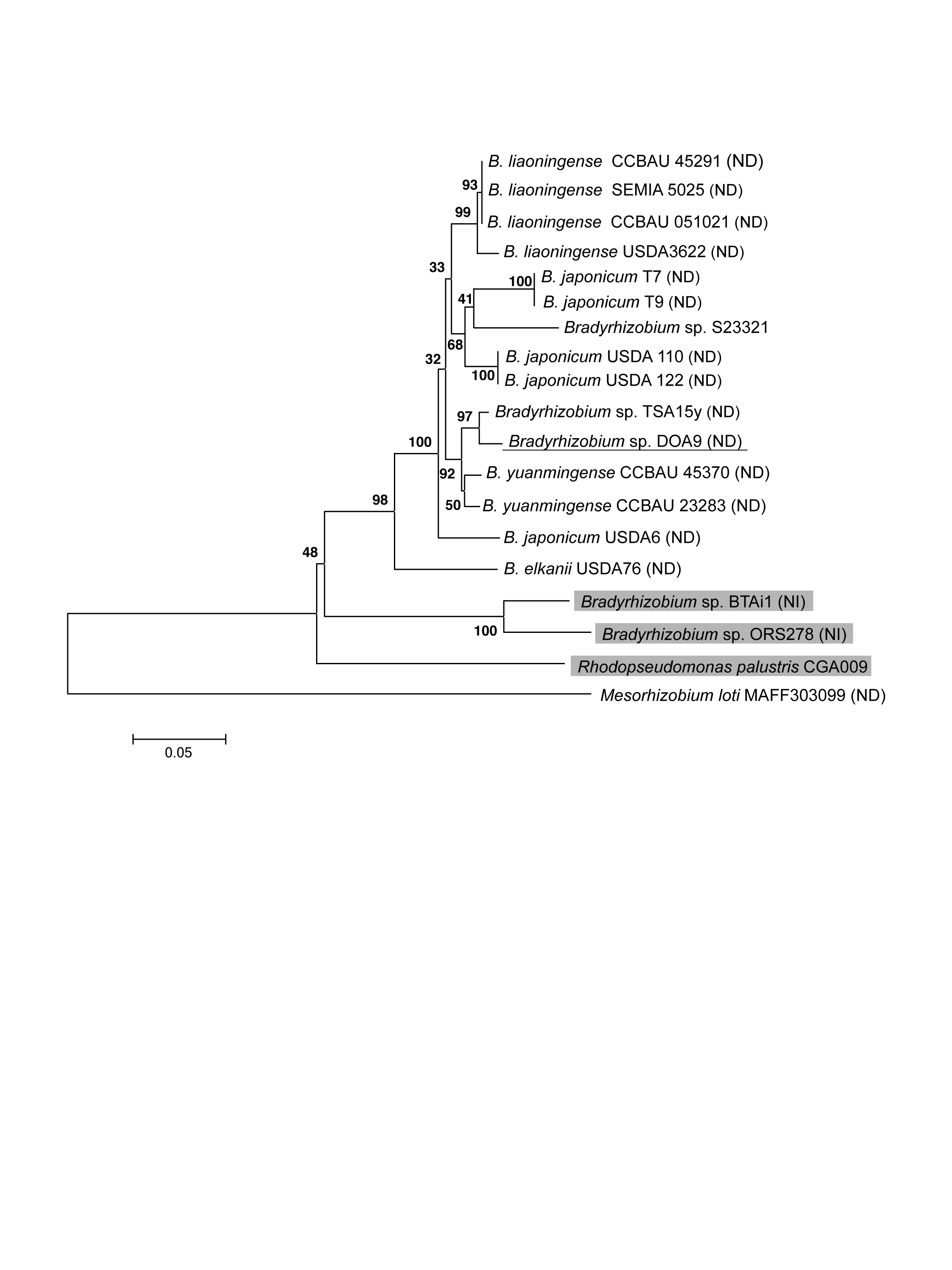

Supplement: S1 Fig — Bootstrap values are expressed as percentages of 1,000 replications. Evolutionary distances were computed using the Kimura two-parameter method. The bar represents one estimated substitution per 100-nucleotide positions. Strains capable of Nod factor-dependent and -independent nodulation are marked with (ND) and (NI), respectively. Photosynthetic strains are highlighted in gray. (TIF) [file pone.0117392.s001.tif]

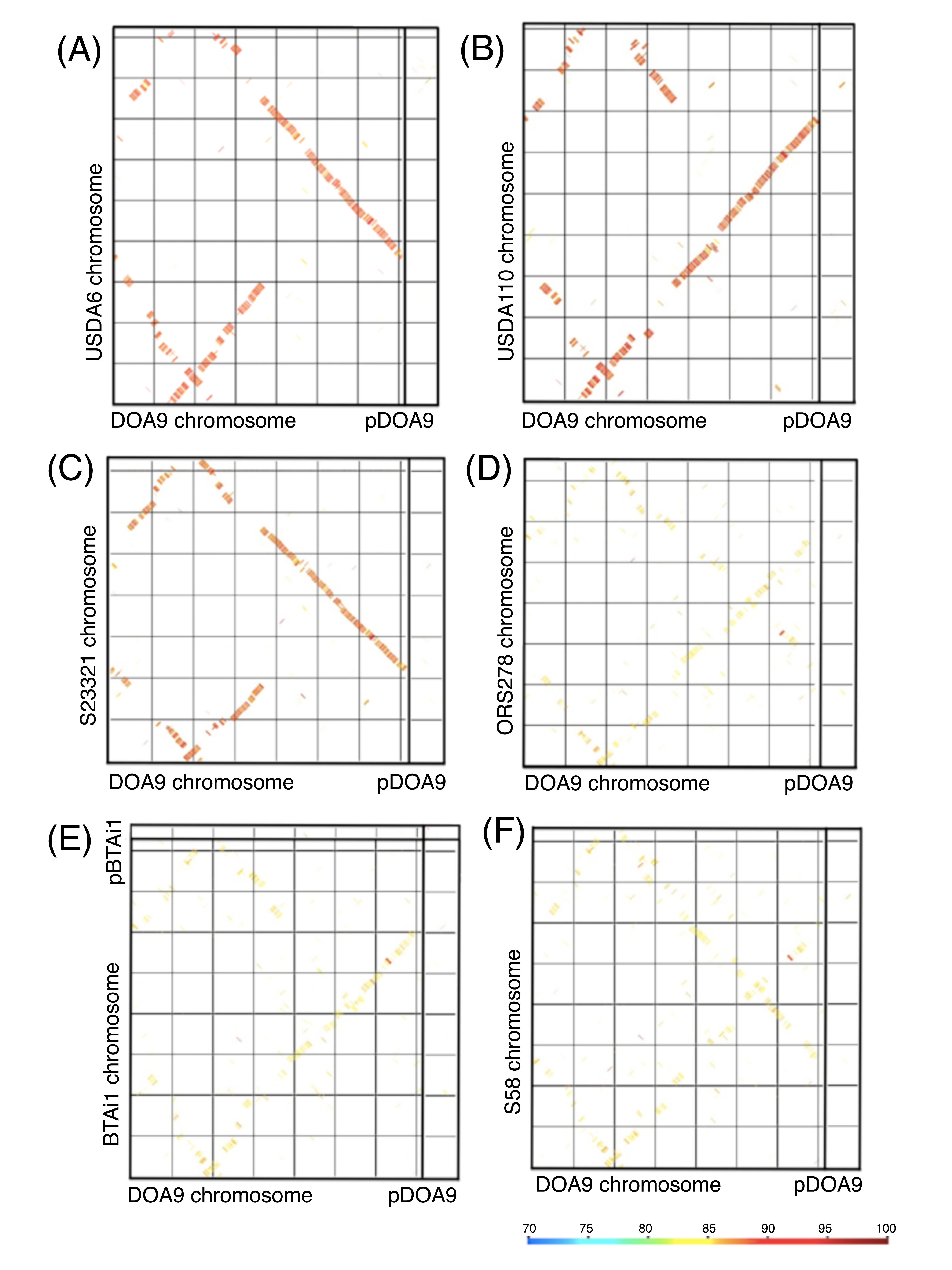

Supplement: S2 Fig — The positions on each chromosome of each bradyrhizobial strain are indicated on the x-axis and y-axis. The dot color indicates the percentage similarity, as indicated in the key. (TIF) [file pone.0117392.s002.tif]

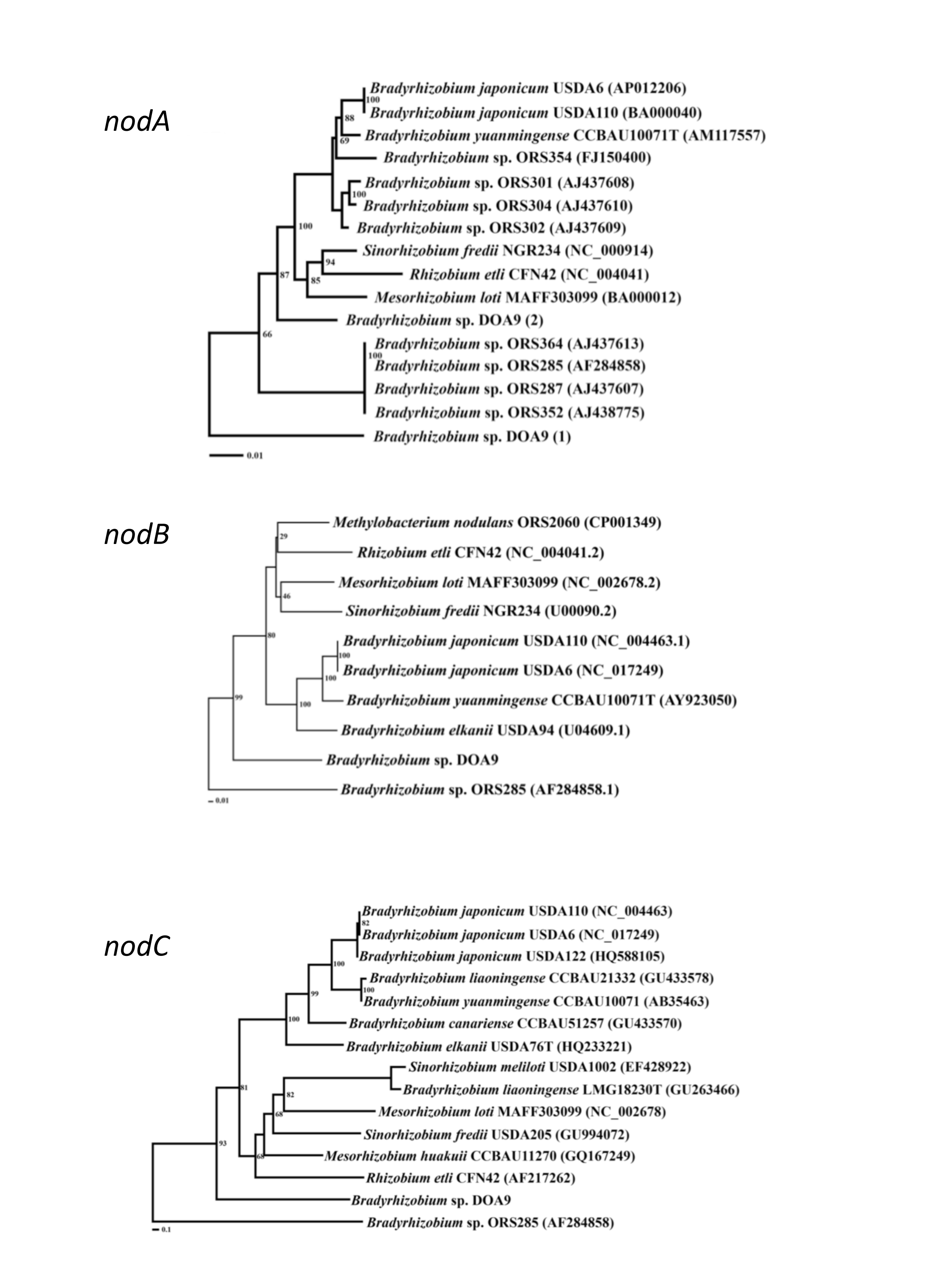

Supplement: S3 Fig — Bootstrap values are expressed as percentages of 1,000 replications. The bar represents one estimated substitution per 100-nucleotide positions. (TIF) [file pone.0117392.s003.tif]

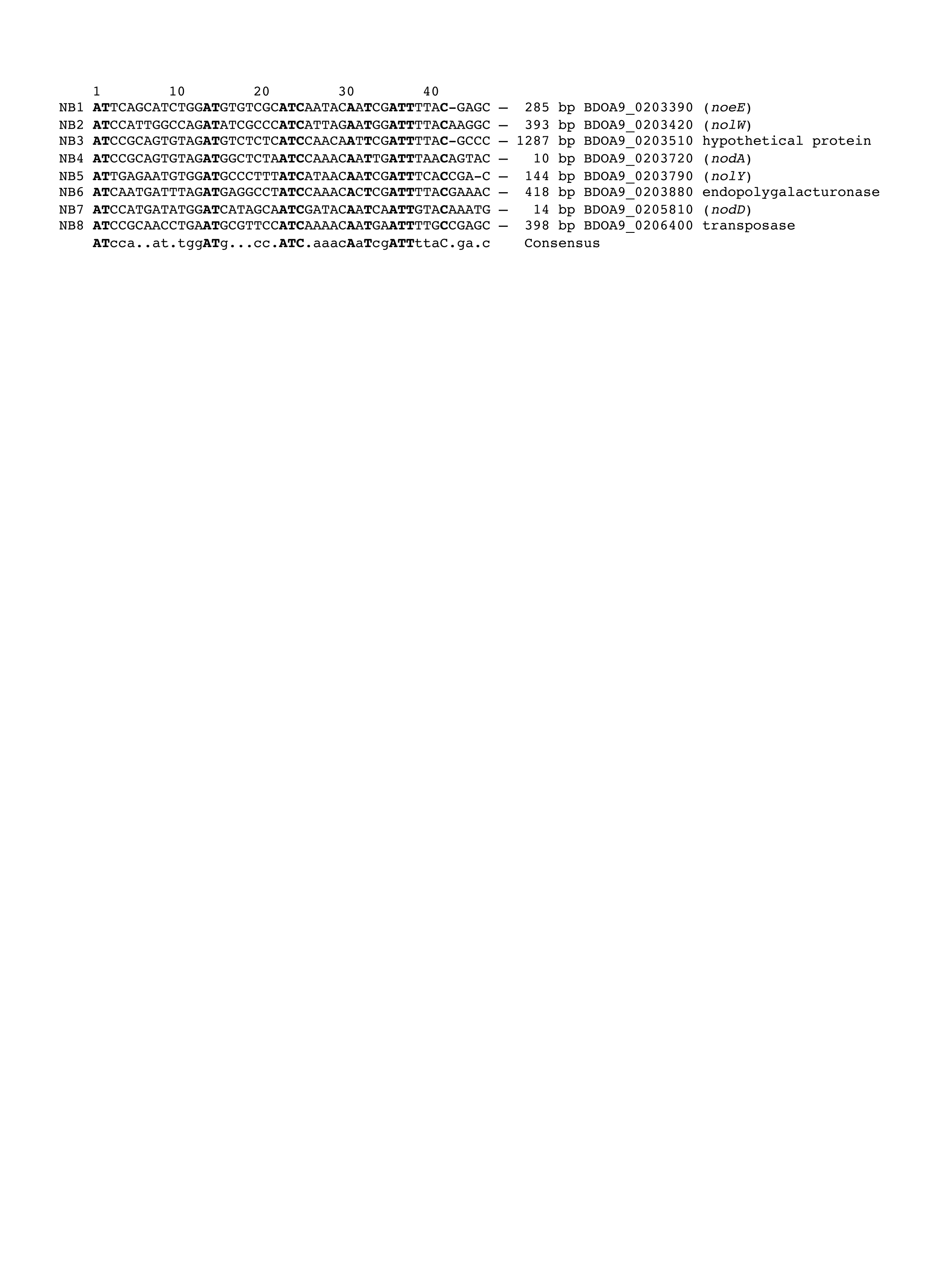

Supplement: S4 Fig — Nucleotides conserved in all cases are shown in bold uppercase letters. Numbers indicate the distance in base pairs between the nod box and the potential translational start site of the corresponding gene. In the consensus sequence capital letters are used for invariant nucleotides, and lowercase letters are used for nucleotides conserved in at least 50% of the sequences. (TIF) [file pone.0117392.s004.tif]

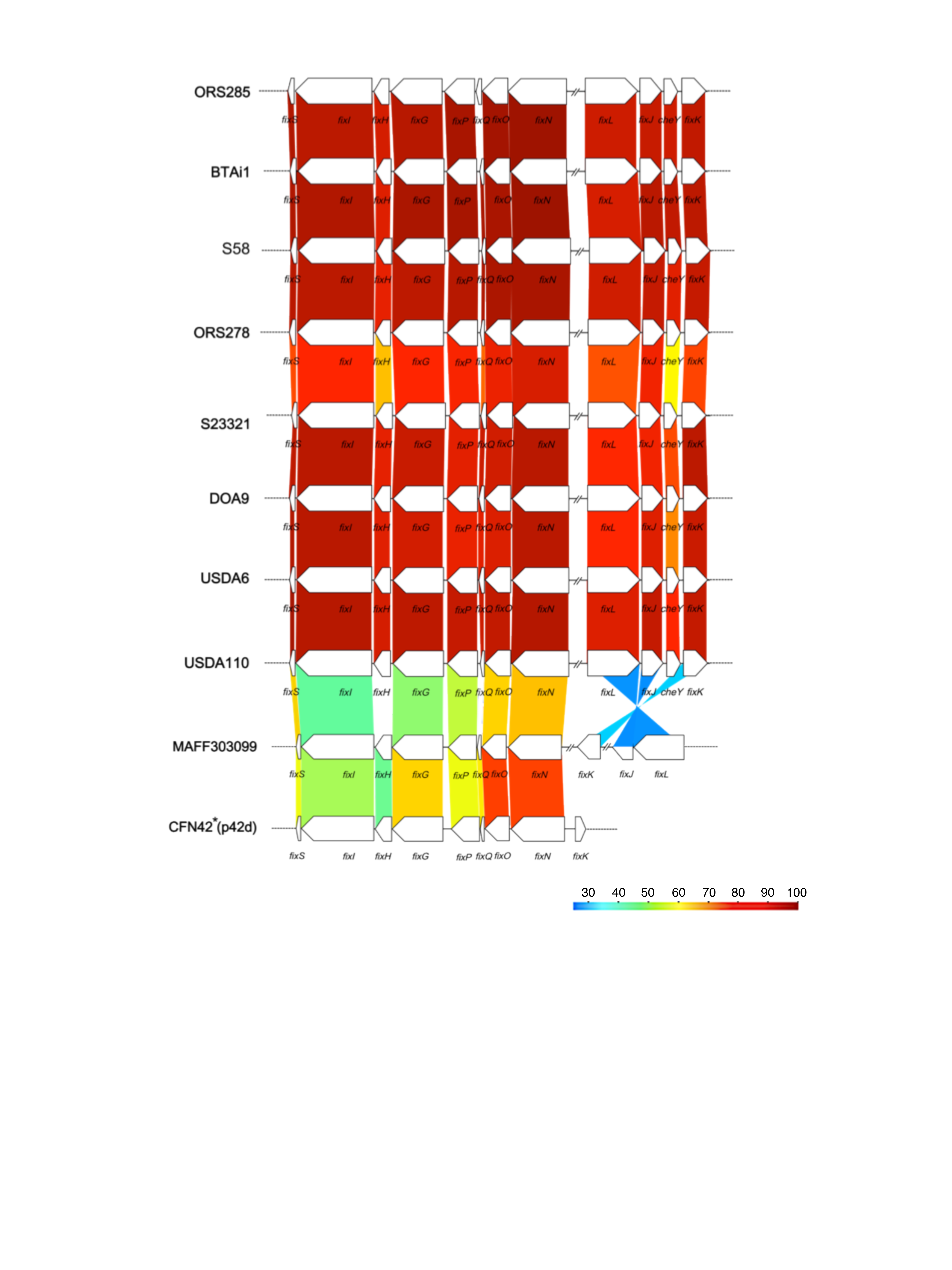

Supplement: S5 Fig — Double slash marks represent DNA regions that are not shown. Colored strips represent the conserved gene regions between the compared strains, and the color indicates the percentage similarity, as indicated by the key. (TIF) [file pone.0117392.s005.tif]

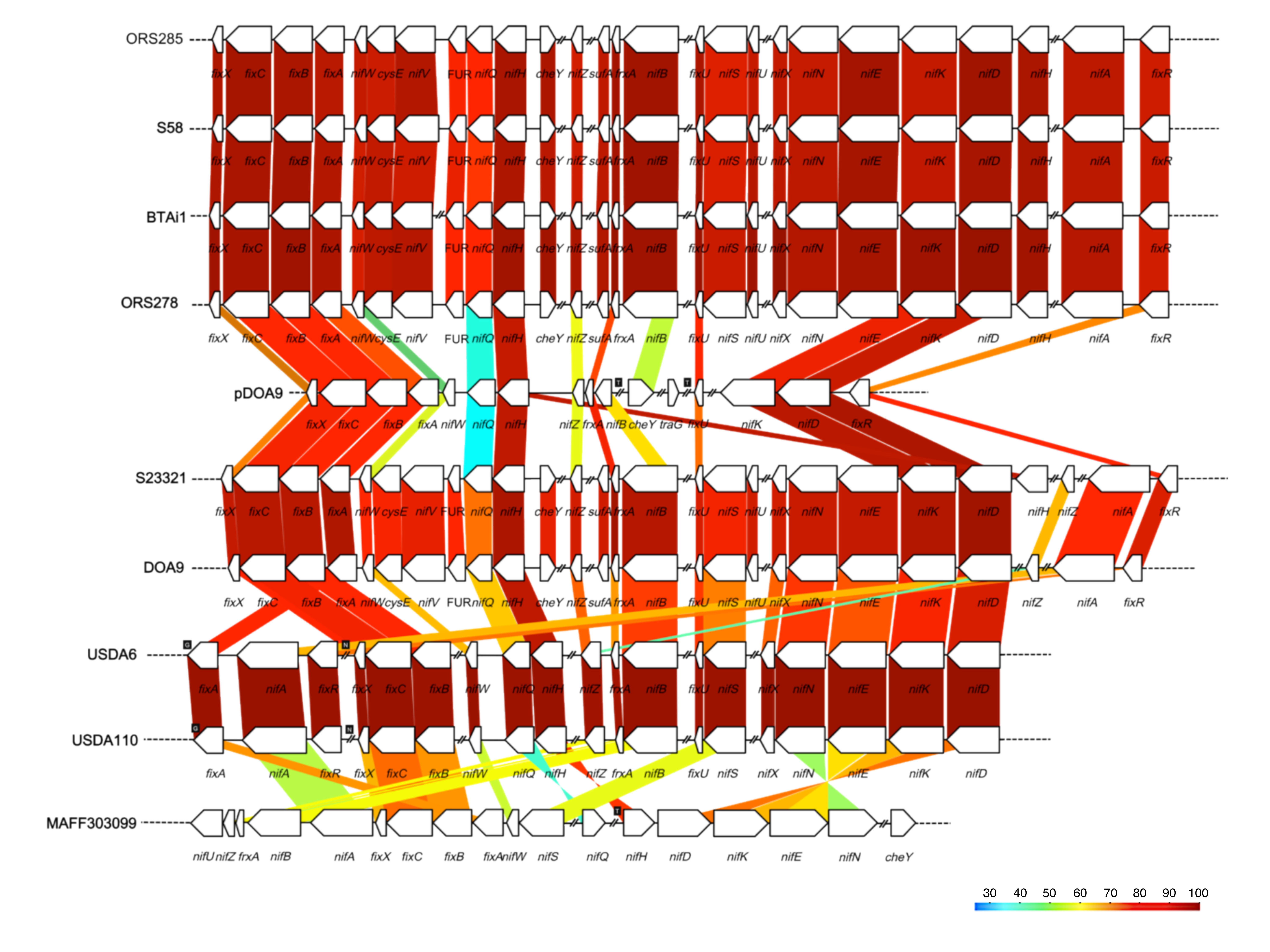

Supplement: S6 Fig — Double slash marks represent DNA regions that are not shown. Colored strips represent the conserved gene regions between the compared strains, and the color indicates the percentage similarity, as indicated by the key. T: region where the transposase genes were located. N: region of nodulation genes. G: region of groES-groEL regulatory genes. (TIF) [file pone.0117392.s006.tif]

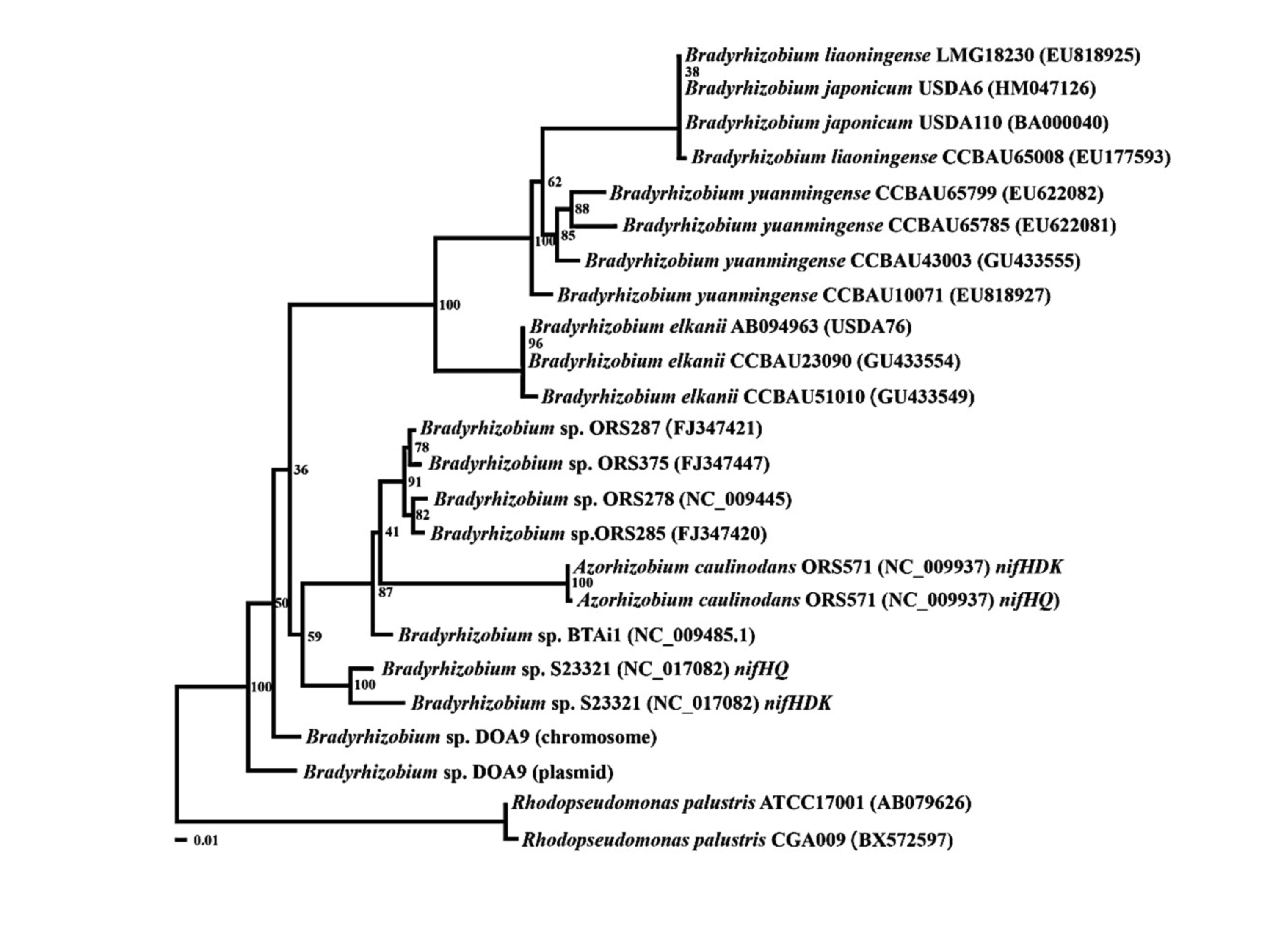

Supplement: S7 Fig — Bootstrap values are expressed as percentages of 1,000 replications. The bar represents one estimated substitution per 100-nucleotide positions. (TIF) [file pone.0117392.s007.tif]

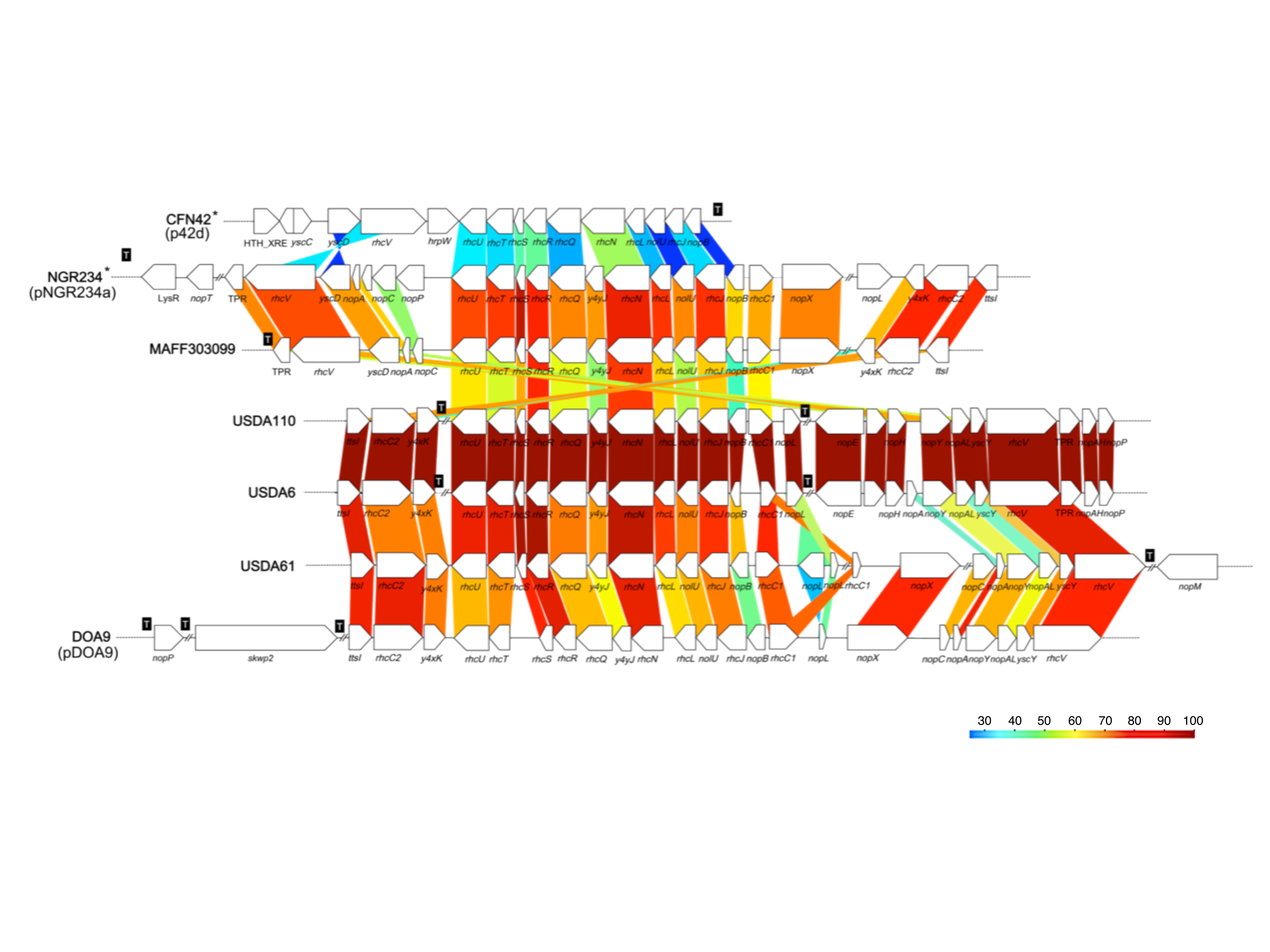

Supplement: S8 Fig — Asterisks represent the strains harboring the T3SS cluster in the plasmid and each plasmid name is shown in parentheses. Double slash marks represent DNA regions that are not shown. Colored stripes represent the conserved gene regions between the compared strains, and the color indicates the percentage similarity, as indicated by the key. T: region where the transposase genes were located. (TIF) [file pone.0117392.s008.tif]

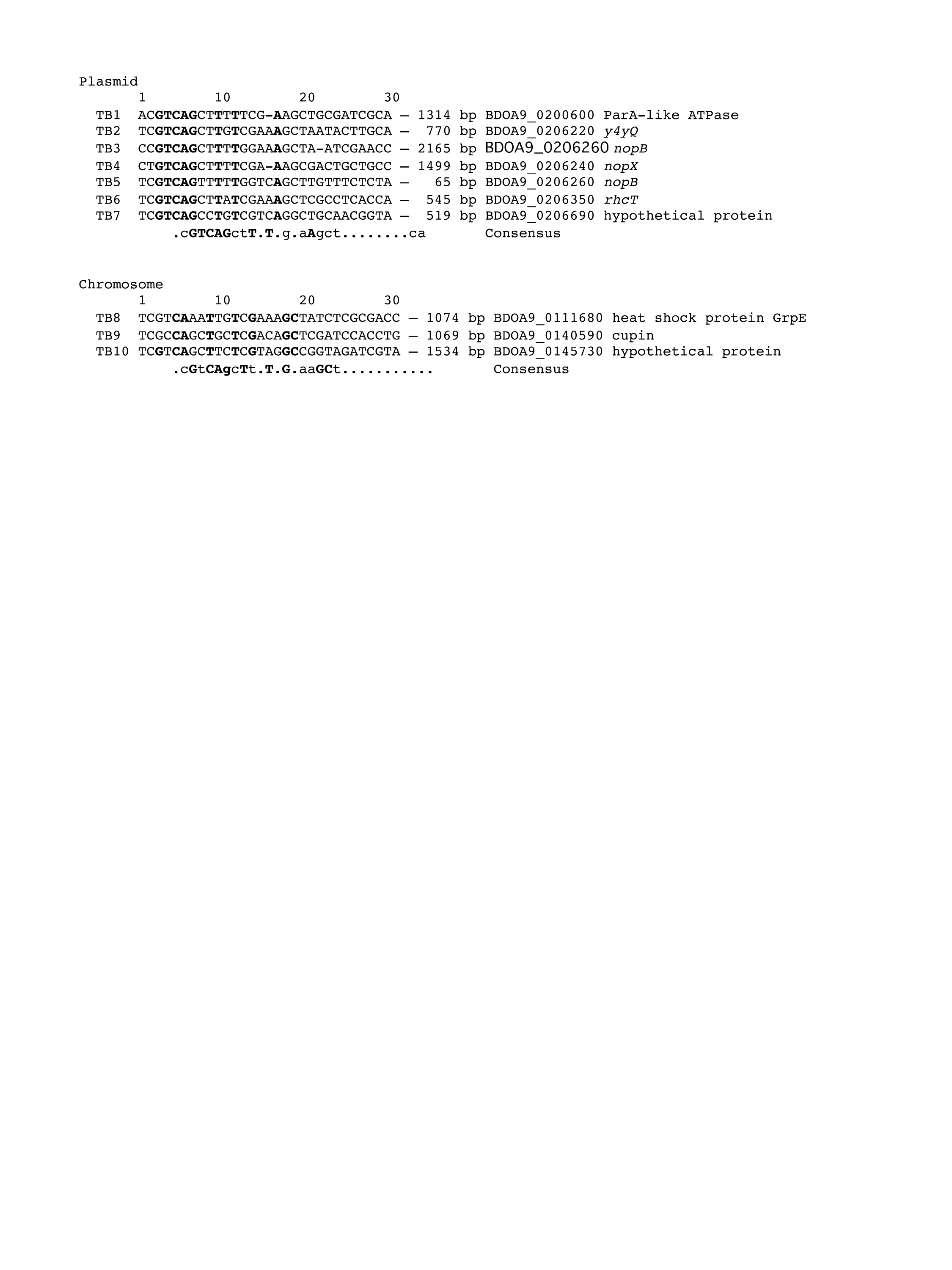

Supplement: S9 Fig — Nucleotides conserved in all cases are shown in bold uppercase letters. Numbers indicate the distance in base pairs between the tts box and the potential translational start site of the corresponding gene. In the consensus sequence capital letters are used for invariant nucleotides, and lowercase letters are used for nucleotides conserved in at least 50% of the sequences. (TIF) [file pone.0117392.s009.tif]

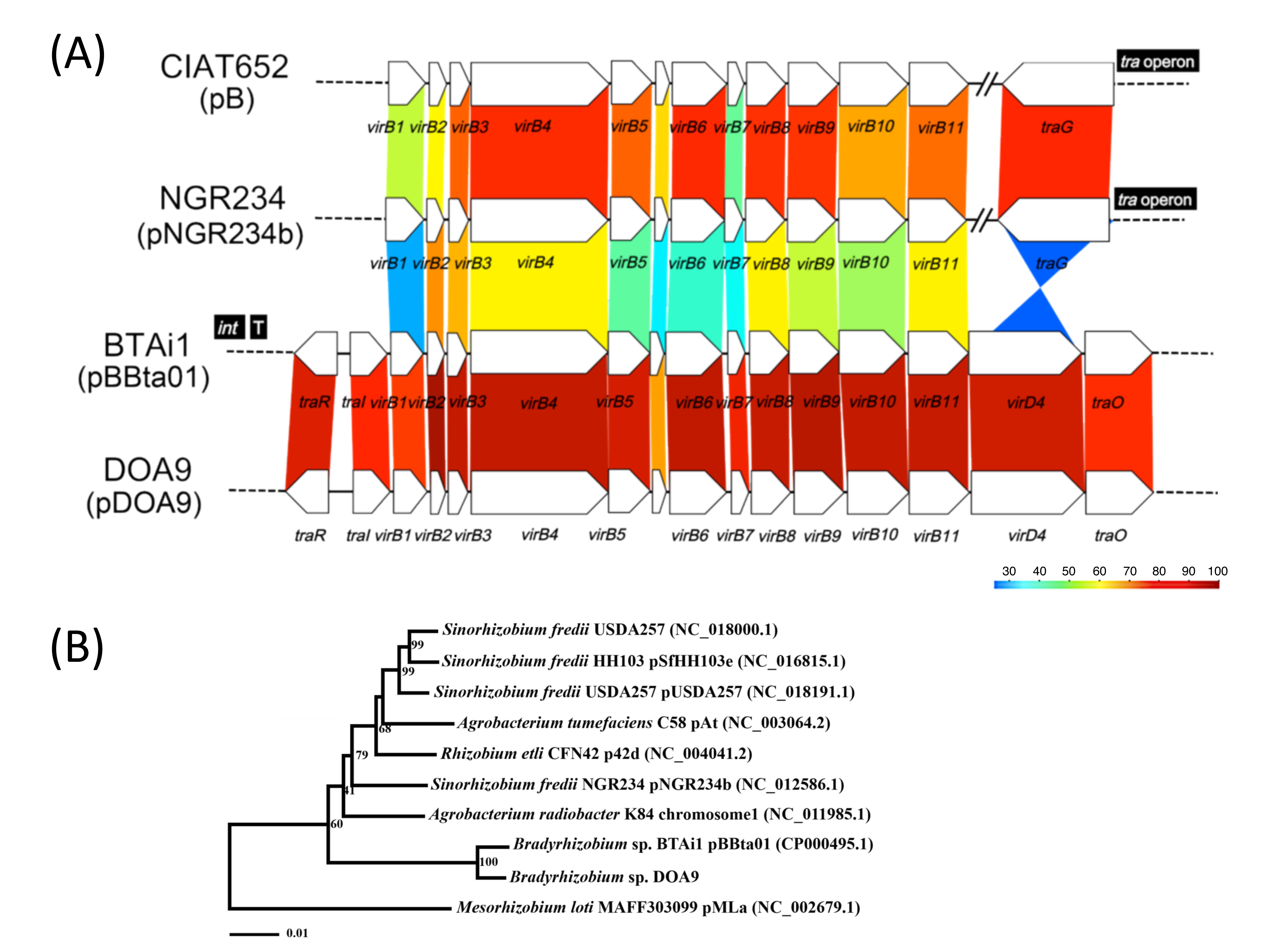

Supplement: S10 Fig — (A) Comparative analysis of vir clusters of Bradyrhizobium sp. DOA9 and related bacteria. All the compared clusters were located on the symbiotic plasmid and each plasmid name is shown in parentheses. Double slash marks represent DNA regions that are not shown. Colored strips represent the conserved gene regions between the compared strains, and the color indicates the percentage similarity, as indicated by the key. (B) Phylogenetic trees based on a combination of the virB2 and virB9 sequences of DOA9 and other related rhizobia. Bootstrap values are expressed as percentages of 1,000 replications. The bar represents one estimated substitution per 100-nucleotide positions. (TIF) [file pone.0117392.s010.tif]
